# Biological Control of Melon Continuous Cropping Obstacles: Weakening the Negative Effects of the Vicious Cycle in Continuous Cropping Soil

**DOI:** 10.1128/spectrum.01776-22

**Published:** 2022-10-27

**Authors:** Yongli Ku, Wenqiang Li, Xueli Mei, Xiangna Yang, Cuiling Cao, Huimei Zhang, Le Cao, Minglei Li

**Affiliations:** a College of Life Sciences, Northwest A&F University, Yangling, Shaanxi Province, China; b College of Horticulture, Northwest A&F University, Yangling, Shaanxi Province, China; c College of Environment and Life Sciences, Weinan Normal University, Weinan, Shaanxi Province, China; d Institute of Soil and Water Conservation, Northwest A&F University, Yangling, Shaanxi Province, China; Pennsylvania State University

**Keywords:** phenolic acid, *Fusarium*, root-knot nematode, continuous cropping obstacle, *Bacillus subtilis*

## Abstract

The continuous cropping obstacles of melons have become increasingly serious in recent years. To investigate this, we explored the effects and mechanisms of Bacillus subtilis C3 in control of the continuous cropping obstacles of melon. We provide a novel interaction model of the occurrence factors of continuous cropping obstacles. The dominant pathogen isolated from melon soil was Fusarium. Their hyphae were used as food to cultivate root-knot nematodes. The main phenolic acids in melon soil promoted the growth of Fusarium and indirectly increased the number of root-knot nematodes, but they also had direct toxic effects on melon root-knot nematodes. The simultaneous inoculation of the three had the strongest inhibitory effect on melon seedlings, while the inhibitory effect of paired inoculation was weaker than that of single inoculation. Therefore, the three balance each other, forming a vicious cycle. Bacillus subtilis C3 weakened the negative effects of this cycle on melon by eliminating phenolic acids and inhibiting the growth of Fusarium and root-knot nematodes. Simultaneously, they also alleviated the continuous cropping obstacles of melon by improving the composition and structure of the rhizosphere microbial community. Our results might be useful for the effective control of the continuous cropping obstacles of melon.

**IMPORTANCE** The soil environment, crop growth and fruit quality of melons are negatively affected by long-term continuous cropping. It is important to study the mechanism of continuous cropping obstacles and their biological control. In this study, we propose a novel interaction model of the occurrence factors of continuous cropping obstacles. The dominant phenolic acids, pathogenic fungi, and root-knot nematodes from melon soil balance each other, forming a vicious cycle. Bacillus subtilis C3 weakened the negative effects of this cycle on melon by eliminating phenolic acids and inhibiting the growth of Fusarium and root-knot nematodes. In addition, C3 also improved the composition and structure of the melon rhizosphere microbial community. These results advance the study of the occurrence mechanism of continuous cropping obstacles and demonstrate an efficient and environmentally friendly biological control scheme.

## INTRODUCTION

In recent years, due to the rapid development of facility agriculture, facility soil cultivation has become the main cultivation method for vegetables and fruits. However, due to the single type of facility cultivation, continuous cropping is more common, which seriously restricts the development of facility cultivation technology. The soil environment, crop growth and fruit quality are negatively affected by long-term continuous cropping (1). Melon (*Cucumis melo* L.) is an important facility crop that is widely cultivated (2), and continuous cropping leads to a substantial decrease in its yield by hindering its growth and development.

The plant rhizosphere soil environment is complex and changeable, and the occurrence and aggravation of continuous cropping obstacles are the result of the combined actions of many factors, such as autotoxicity, pathogens, and root-knot nematodes (RKNs) (3, 4). As plants are continuously planted, the root system secretes a complex mixture of phenolic acids, sugars, and free amino acids into the rhizosphere environment that directly or indirectly poison the plant (5). Also, phenolic acids are the main toxic substances in root exudates that are harmful to horticultural crops (6). Soil microbial communities play an indispensable role in maintaining the stability and health of soil ecosystems, and their structural imbalance is an important factor in the formation of continuous cropping obstacles (7). Continuous planting reduces the bacterial diversity in the soil and increases the abundance of pathogenic fungi (8). In addition, continuous cropping leads to an increase in soil RKN populations, and RKNs infect root tissues, thereby reducing their ability to absorb and transport nutrients and water, which damages the host (9). Therefore, the occurrence of continuous cropping obstacles is caused by many factors, but it is unclear whether the effects of these factors are additive or inhibitory.

The main occurrence factors of continuous cropping obstacles coexist, and the relationship between the two of them has been reported. For example, phenolic acids secreted by the root system can directly affect the biological activity of soil microbes, especially the pathogenic fungi (10, 11). Phenolic acids also have toxic effects on RKNs, such as p-coumaric acid, p-hydroxybenzoic acid, cinnamic acid, and ferulic acid (12–14). Additionally, RKNs can feed on pathogenic fungi, and some researchers used Fusarium oxysporum f.sp. *cubense*, Fusarium solani, and Botrytis cinerea to successfully culture RKNs *in vitro* (15, 16). However, the relationship between the three main factors has not been reported, we speculate that there may be a delicate connection among phenolic acids, pathogenic fungi, and RKNs.

Many methods, such as soil sterilization (17), crop rotation (18), and grafting technology (19) have been applied to mitigate continuous cropping obstacles. However, soil sterilization always leads to environmental damage. Crop rotation takes a long time to repair the soil, and pathogenic bacteria and autotoxic substances may still affect the next crop (20). Grafting technology wastes labor and extremely tests the skills of workers. These strategies are not as good as biological control methods that use biocontrol bacteria. Some bacteria, such as *Streptomyces*, *Bacillus*, *Rhizobium*, *Corynebacterium*, and Pseudomonas, have been successfully used to prevent continuous cropping obstacles (5, 21, 22). The application of arbuscular mycorrhizal fungi (AMF) improves the growth of continuous American ginseng by modifying rhizosphere microorganisms that facilitate nutrient acquisition and by inhibiting soilborne pathogens (23). In a previous study, the tomato RKN disease index decreased by 37% after inoculation with *Streptomyces* (24). Another study indicated that Paenibacillus polymyxa CP-S316 alleviates the continuous cropping effect of poplar by regulating the rhizosphere microbial community structure (25). Although the biocontrol method has been applied in alleviating continuous cropping obstacles, there have been only a few studies on its control mechanism.

At the present time, we already know the main factors that lead to the occurrence of continuous cropping obstacles. However, the occurrence mechanism of continuous cropping obstacles and the mechanism of their biological control are still unclear. We hypothesize that there is some kind of connection between the main causes of continuous cropping obstacles and that they promote each other, inhibit each other, or maintain a balance. Also, the biological control method may alleviate the continuous cropping obstacles by weakening the effects of various factors or by destroying the aforementioned connection. In this study, the rhizosphere soil of continuous cropping melon was taken as the research object, and the occurrence mechanism of the continuous cropping obstacles of melon and the control mechanisms of Bacillus subtilis C3 were discussed. Our aims were: (a) to identify the factors that cause continuous cropping obstacles of melon as well as the relationships between them and (b) to determine the effect and mechanism of the biological control of melon continuous cropping obstacles. The overall results of this study provide an interaction model of the occurrence factors of the continuous cropping obstacles of melon and demonstrate how an efficient and environmentally friendly biological control scheme involving C3 can work.

## RESULTS

### Isolation and identification of pathogenic fungi in the soil of continuously cropped melon soil.

**FIG 1:**
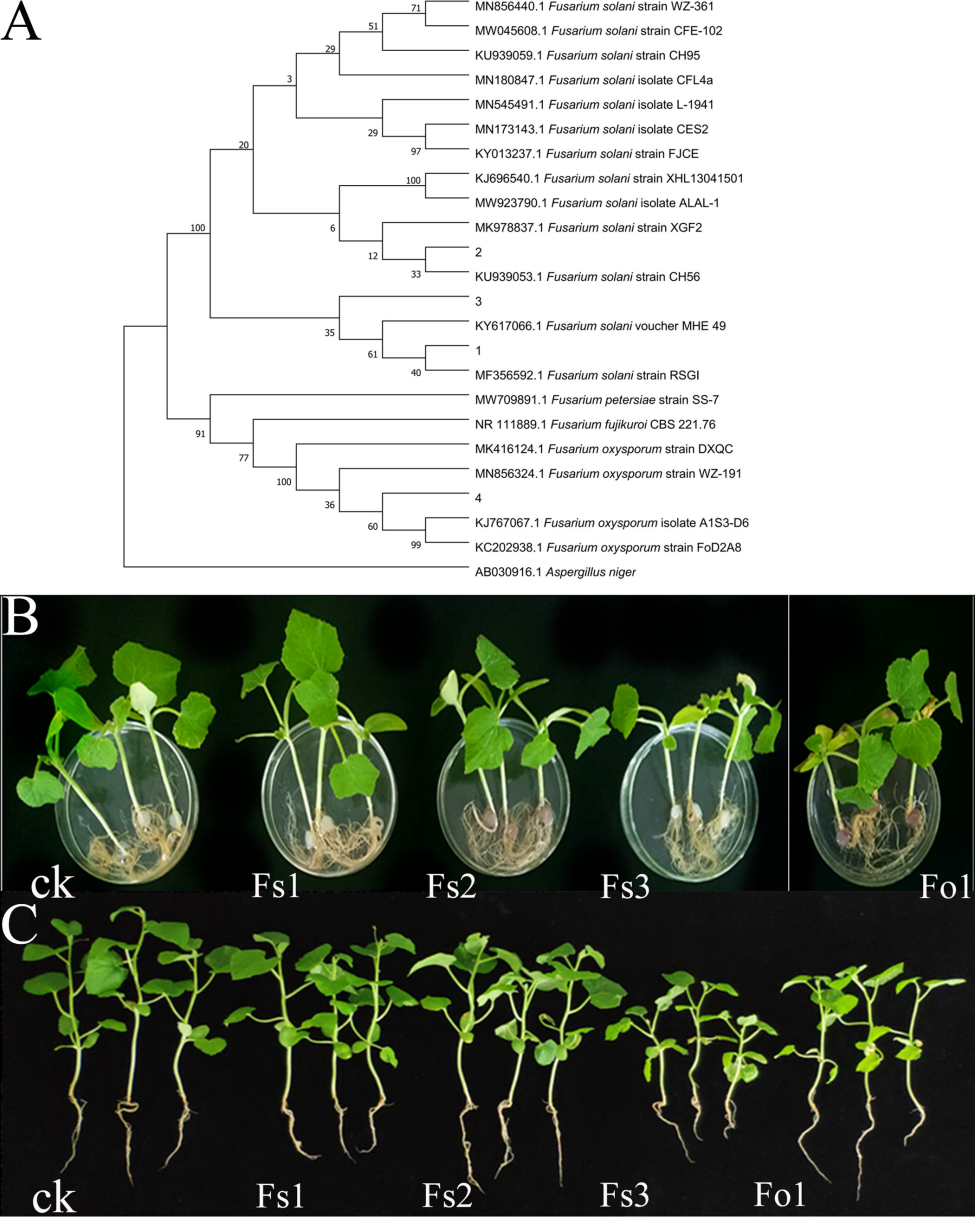
Identification of pathogenic fungi in continuous melon cropping soil. (A) Phylogenetic tree analysis of four kinds of fungi. (B) The indoor fungi plug reinoculation experiment. (C) The pot experiment, in which four strains of a suspension of Fusarium spores were sprinkled on the roots of melon seedlings.

The dilution coating method was used to isolate eight fungi from the rhizosphere soil of melon that had been cropped continuously for 3 years. After morphological observation, they were determined to belong to the genera Fusarium (4), Aspergillus (3) and *Mucor* (1) (Fig. S1). Four strains of Fusarium were isolated from rhizosphere soil that had been continuously cropped with melon and numbered from 1 to 4. The 4 strains of the Fusarium colonies were all white on the front, the second Fusarium was yellowish-brown on the reverse side, and the fourth Fusarium produced a purple water-soluble pigment and a dark purple color on the reverse side (Fig. S1A–D). The four strains of Fusarium mycelium had septa and branches (Fig. S1a–d). Their conidia presented as large and small. The small conidia were oval or cylindrical with 1 to 2 septa, and the large conidia were sickle-shaped or long and cylindrical with many septa (Fig. S1e–h). The measured sequence lengths of the ITS regions of the four Fusarium strains were 566 bp, 553 bp, 563 bp, and 543 bp, respectively. Subsequently, the ITS sequences of the tested strains were compared with those of related strains in GenBank. The constructed phylogenetic tree showed that strains 1, 2, and 3 (Fs1, Fs2, and Fs3) were the three strains of Fusarium solani and that strain 4 (Fo1) was a strain of Fusarium oxysporum (Fig. 1A).

In an indoor fungal plug reinoculation experiment, after inoculation with fungal plugs at the junction of the shoot and roots (one fungal plug per seedling), the pathogenic effect of Fs3 was the most obvious. The melon seedling leaves withered, and the roots browned 5 d after inoculation with Fs3, while the Fo1-treated leaves began to wilt and turn yellow; however, inoculation with Fs1 and Fs2 had no obvious effect on the melon seedlings (Fig. 1B). In a pot experiment, spore suspensions of four strains of Fusarium were sprinkled on the roots of melon seedlings (10 mL/plant, 10^6^ spores/mL). Fs1 inhibited the growth of the melon seedlings, although the differences in biomass and agronomical traits did not significantly differ compared with a control. In addition, Fs2 had no effect on the growth of melon seedlings, whereas Fs3 and Fo1 infected the root system of melon seedlings and significantly inhibited the growth (*P* < 0.05). The inhibitory effect of Fs3 was the most obvious (Fig. 1C; Table S2).

### Interaction of Fusarium, phenolic acids, and RKNs in continuous melon cropping soil.

**FIG 2:**
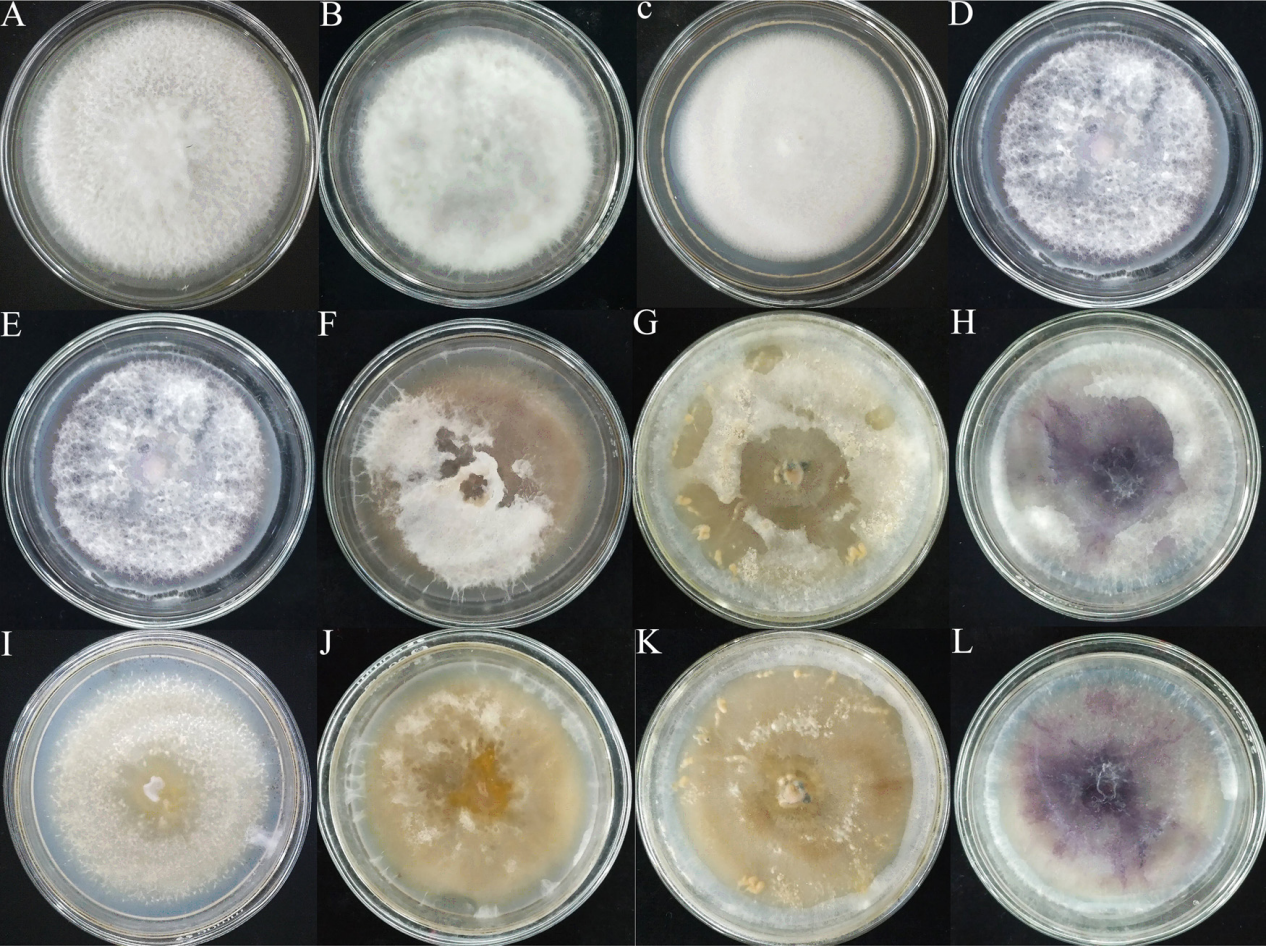
Effects of cocultivation of Fusarium and RKNs. (A–D) Fusarium Fs1, Fs2, Fs3, and Fo1 double medium. (E–H) Fs1, Fs2, Fs3, and Fo1 double medium inoculated with RKNs after 14 d. (I–L) Fs1, Fs2, Fs3, and Fo1 double medium inoculated with RKNs after 21 d.

Fusarium Fs1, Fs2, Fs3, and Fo1 double culture medium mycelium gradually disappeared with the extension of cultivation time, indicating that RKNs can consume mycelia and that these four strains of Fusarium can be used to prepare double culture medium to culture RKNs *in vitro* (Fig. 2). After 21 d of culture, double culture medium agar plugs with a diameter of 6 mm were collected, and the density of RKNs was counted under a microscope. The density of the RKNs in the Fo1 double medium was the highest and exceeded 12,000/cm^3^, followed by that of Fs2, which reached 10,000/cm^3^. The density of the RKNs in the Fs3 double medium reached 9,000/cm^3^ (Table S3).

After drying and weighing the mycelium filtered with filter paper, we found that coumaric acid (CA), ferulic acid (FA), and *p*-hydroxybenzoic acid (HA) at 5, 10, and 20 mg/L all had a promoting effect on the growth of Fusarium Fs3 mycelium, with the best promoting effects of 13.4%, 12.6%, and 13.9% occurring at 10 mg/L, respectively (Table 1).

**TABLE 1:** Effect of different concentrations of phenolic acids on the growth of Fs3 mycelium and a toxicity analysis to RKNs^*a*^

Phenolic acids	Concentration (μg/L)	Mycelium dry weight (mg)	Promotion rate (%)	Concentration (mg/L)	Mortality rate (%)	Adjusted mortality rate (%)
CK	0	79.333 ± 2.517b		0	13.333	
CA	5	81.333 ± 3.215ab	2.5	25	17.391	4.683
	10	90.000 ± 0.000a	13.4	50	27.586	16.446
	20	83.667 ± 6.351ab	5.5	100	30.769	20.119
				200	43.478	34.783
				400	48.000	40.000
FA	5	87.000 ± 4.583ab	9.7	25	15.789	2.834
	10	89.333 ± 6.506a	12.6	50	19.444	7.052
	20	87.667 ± 3.215ab	10.5	100	33.333	23.077
				200	40.000	30.769
				400	45.833	37.500
HA	5	89.333 ± 4.041a	12.6	25	26.316	14.980
	10	90.333 ± 4.041a	13.9	50	43.333	34.616
	20	86.000 ± 6.403ab	8.4	100	46.429	38.187
				200	62.500	56.731
				400	74.194	70.223

a Mean data with different lowercase letters indicate a statistically significant difference (*P* < 0.05).

After treatment with different concentrations of CA, FA, and HA solutions for 48 h, different concentrations of the three phenolic acids had different degrees of toxicity with respect to the RKNs. At 25 to 400 μg/L, CA and FA had no difference in RKN lethality. HA was more toxic to RKNs than were CA and FA, and the corrected mortality rate at 400 μg/L was 12.467%. When the concentration was 25 to 400 mg/L, the lethal rate of CA, FA, and HA to the RKNs increased with the concentration, although, when at the same concentration, HA was more toxic to the RKNs. When the HA concentration was 400 mg/L, the corrected RKN mortality was the highest (70.223%) (Table 1). In short, HA was the most toxic to RKNs, followed by FA, and CA was the least toxic.

### Inhibitory effects of HA, Fusarium, and RKNs on the growth of melon seedlings.

Inoculation with HA, Fs3, and RKNs inhibited the growth of melon. Among them, the inhibitory effect of RKNs alone was higher than that of HA or FS3 alone. The inhibitory effects of Fs3 + HA and RKN + Fs3 not only were weaker than those of RKN + HA but also were weaker than those of Fs3 and RKN alone. Compared with the control (CK), the combination of two factors (Fs3 + HA, RKN + Fs3, RKN + HA) had weaker effects than the single inoculations. Inoculating the three together (RKN + Fs3 + HA) simulated a continuous cropping environment for melon and had the most significant inhibitory effect on melon growth (*P* < 0.05). Compared with the control, the plant height, root length, leaf number, shoot fresh weight, shoot dry weight, root fresh weight, and root dry weight were reduced by 45.3%, 19.6%, 31.5%, 53.3%, 67.4%, 50.0%, and 57.1%, respectively (Fig. S2; Table 2).

**TABLE 2:** Effect of C3 on the biomass and horticultural traits of melons inoculated with HA, Fs3, and RKNs^*a*^

Treatments	Plant height (cm/plant)	Root length (cm/plant)	Number of leaves (per plant)	Shoot fresh weight (g/plant)	Root fresh weight (g/plant)	Shoot dry weight (g/plant)	Root dry weight (g/plant)	Plant fresh weight (g/plant)	Plant dry weight (g/plant)
CK	48.1 ± 2.5a	15.8 ± 0.7ab	7.3 ± 0.6a	6.562 ± 0.382a	0.298 ± 0.015c	0.473 ± 0.028a	0.021 ± 0.001b	6.860 ± 0.379a	0.487 ± 0.026a
RKN	30.6 ± 2.3g	12.7 ± 1.7c	5.6 ± 0.5d	3.342 ± 0.118g	0.190 ± 0.019ef	0.211 ± 0.019e	0.012 ± 0.002ef	3.465 ± 0.149e	0.222 ± 0.021d
Fs3	34.9 ± 2.4ef	13.5 ± 1.1c	6.6 ± 0.5bc	4.635 ± 0.322def	0.197 ± 0.020ef	0.268 ± 0.004d	0.013 ± 0.001de	4.650 ± 0.477cd	0.281 ± 0.004c
HA	37.8 ± 2.4de	12.4 ± 0.6c	6.6 ± 0.5bc	4.809 ± 0.274cde	0.213 ± 0.013de	0.346 ± 0.006c	0.017 ± 0.001bcd	5.026 ± 0.259c	0.361 ± 0.008b
Fs3+HA	37.7 ± 2.9de	13.7 ± 0.6c	6.8 ± 0.4abc	4.865 ± 0.239cd	0.170 ± 0.012fg	0.346 ± 0.018c	0.011 ± 0.001ef	5.071 ± 0.261c	0.357 ± 0.017b
RKN+Fs3	37.2 ± 2.7de	14.0 ± 1.8bc	6.6 ± 0.5bc	4.229 ± 0.136f	0.157 ± 0.027g	0.314 ± 0.006cd	0.018 ± 0.004bc	4.409 ± 0.117d	0.326 ± 0.011bc
RKN+HA	33.3 ± 1.8fg	13.0 ± 0.6c	6.3 ± 0.5c	4.357 ± 0.459ef	0.165 ± 0.009fg	0.297 ± 0.030d	0.017 ± 0.001cd	4.521 ± 0.456cd	0.311 ± 0.029c
RKN+Fs3+HA	26.3 ± 2.7h	12.7 ± 1.6c	5.0 ± 0.0e	3.065 ± 0.329g	0.149 ± 0.007g	0.154 ± 0.016f	0.009 ± 0.002f	3.240 ± 0.354e	0.162 ± 0.018e

a Mean data with different lowercase letters indicate a statistically significant difference (*P* < 0.05).

### Mechanism of Bacillus subtilis C3 in preventing continuous cropping obstacles.

**(i) Decomposition and utilization of phenolic acids by C3.** When C3 was inoculated on an MB solid plate containing CA, FA, and HA as the carbon source for 7 d, the colony morphology of C3 could be seen (Fig. S3b–d). It could not grow on a medium without a carbon source (Fig. S3a). The results showed that C3 could decompose and use phenolic acids.

**(ii) Inhibitory effect of C3 on the growth of Fusarium hyphae.** In the modified PDB liquid medium, four strains of Fusarium grew normally with a large amount of hyphae, while in the medium inoculated with C3, hyphae growth was significantly inhibited (Fig. S4). After 5 d of inoculation, the inhibition rate of C3 on the growth of melon pathogenic Fusarium Fs3 mycelia was 37.580%. Among the other three fungi, it was also over 25% (Table S4). The results showed that C3 could inhibit the growth of Fusarium.

**FIG 3:**
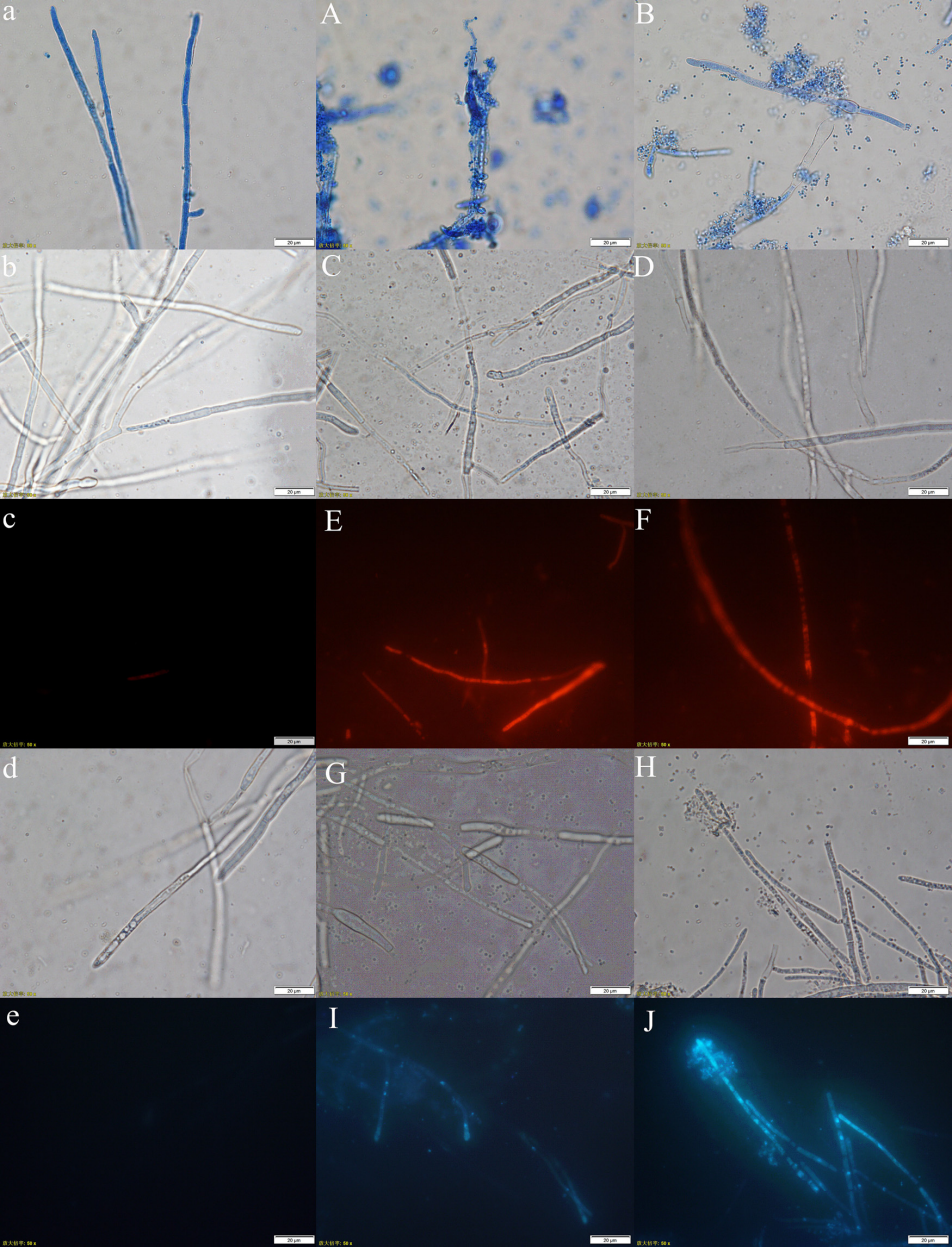
Inhibitory effect of C3 on Fusarium growth. (a–e) Control. (A and B) Lacto phenol cotton blue staining after Fs3 was treated with C3 for 8 h and 24 h. (E and F) PI staining after Fs3 was treated with K3 for 8 h and 24 h. (I and J): Hoechst 33258 staining after Fs3 was treated with C3 for 8 h and 24 h. (C, D, G, and H) Corresponding bright field images. Bars = 20 μm.

After treatment with C3, Fs3 was stained with lactophenol cotton blue, and the mycelium morphology was found to be abnormal. After 8 h of treatment with C3, Fs3 mycelium was deformed, and after treatment with C3 for 24 h, mycelium protoplasts were leaked (Fig. 3A and B). After staining with propidium iodide (PI), a red color of the fungal hyphae under a fluorescence microscope indicated that the cell membrane of the fungal hyphae was destroyed, resulting in the binding of nucleic acid to PI. The untreated Fs3 mycelium could not be stained with PI, while the C3-treated mycelium could be stained with PI. After 8 h of the C3 treatment, only a small part of the Fs3 mycelia was stained, whereas after 24 h, a large amount of mycelium was stained with PI (Fig. 3c, E, and F). The results showed that C3 could destroy the integrity of the mycelial cell membrane, resulting in increased membrane permeability. Hoechst 33258 is often used in the detection of apoptosis. After staining the apoptotic cells and observing them under a fluorescence microscope, a strong blue fluorescence was emitted from the cells. After the Fs3 mycelium was stained with Hoechst 33258, the control mycelium cells had no fluorescence, while the C3-treated mycelium emitted blue fluorescence. After 8 h of treatment with C3, the blue fluorescence was weak, and at 24 h, the fluorescence increased, and the mycelial cells emitting the blue fluorescence increased (Fig. 3e, I, and J).

**(iii) Control effect of C3 on continuous cropping soil RKNs**. After inoculation with RKNs, the root length, maximum leaf area, leaf number, fresh weight of the aboveground part, and fresh weight of the root were reduced by 12.7%, 6.1%, 7.5%, 19.8%, and 29.9%, respectively. The biomass and agronomic traits of the CK and C3 + RKN treatment groups were not only higher than those of the RKN group (*P* < 0.05) but also showed that C3 and its fermented products could promote the growth of melon seedlings (Table S5). The number of RKNs was the highest in the RKN treatment at 680.2 per 100 g of dry soil. The number of RKNs in the C3 + RKN treatment group was 327.1 per 100 g of dry soil, which was 51.91% lower than that observed in the RKN group.

**FIG 4:**
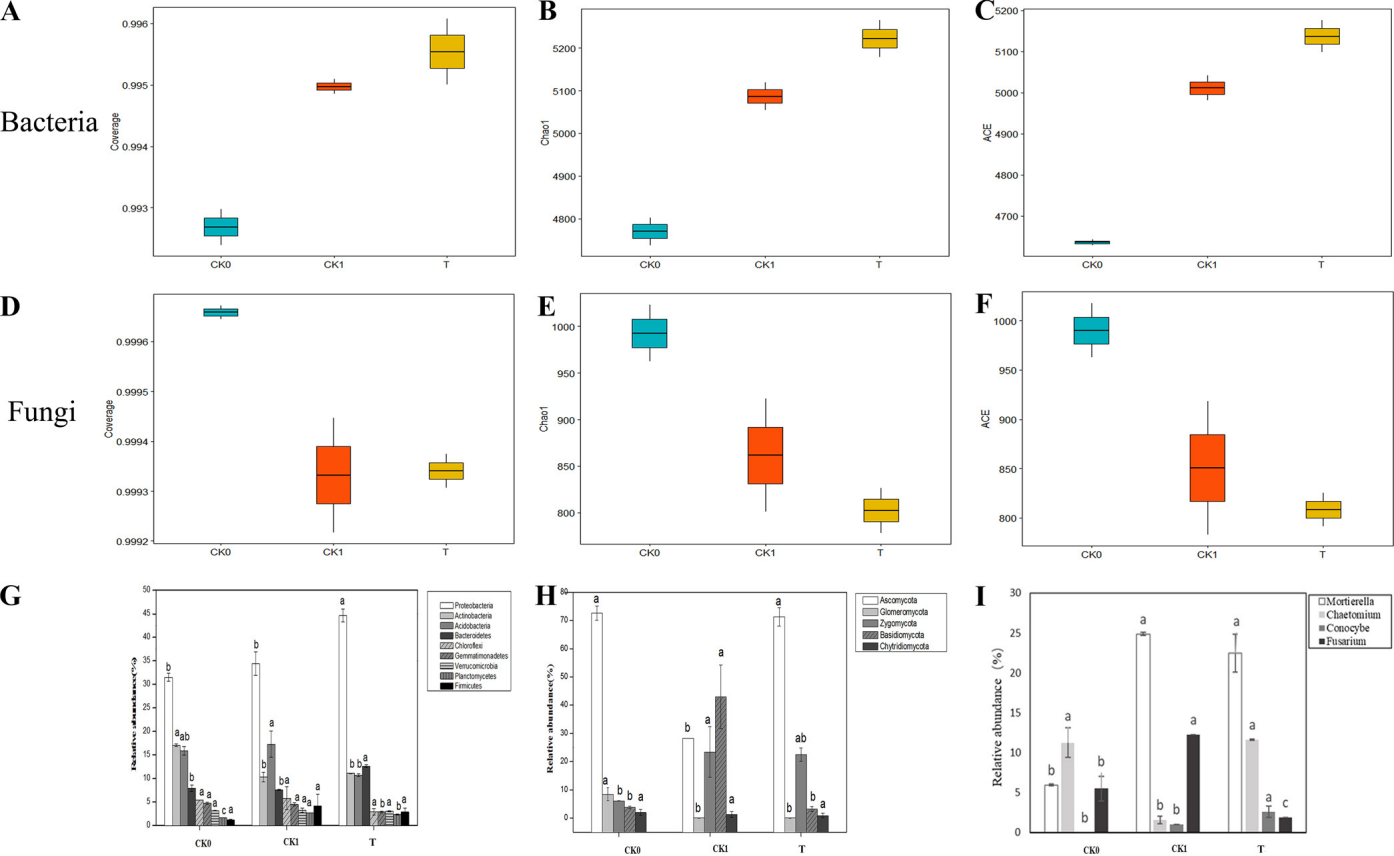
The effect of different treatments on soil microbial communities. CK0, control treatment with healthy soil; CK1, control treatment with monocropping soil; T, High concentration of C3 inoculated treatment with monocropping soil (3 g/kg soil). Different lowercase letters among treatments indicate a statistically significant difference (*P* < 0.05). (A–C) Coverage, Chao1 and ACE index of soil bacteria. (D–F) Coverage, Chao1 and ACE index of soil fungi. (G) Community composition of bacteria in different soils at phyla level. (H and I) Community composition of fungi in different soils at phyla and genus level.

**(iv) Improvement of soil microorganisms in continuous cropping by C3.** The number of bacterial sequences per sample ranged from 99,654 to 127,418, with an average of 113,636 ± 14,516 reads. The coverage ranged between 99.22% and 99.28%, demonstrating that most of the bacterial taxa in the soil samples were detected (Fig. 4A). Moreover, higher Chao1 and ACE indices of the T treatment were shown than those measured under CK1 (Fig. 4B and C), indicating that the richness of the bacterial species in the continuous soil increased greatly after treatment with C3. At the phylum level, we focused on phyla with a relative abundance of over 1.0%. Overall, the dominant phyla across all samples were *Proteobacteria* (31.50% to 44.63%), *Actinobacteria* (10.31% to 17.05%), *Acidobacteria* (10.70% to 17.29%), *Bacteroidetes* (7.60% to 12.60%), *Chloroflexi* (2.96 to 5.77%), *Gemmatimonadetes* (2.88% to 4.72%), *Verrucomicrobia* (3.06% to 3.27%), *Planctomycetes* (1.62% to 2.69%), and *Firmicutes* (1.16% to 4.21%). Compared with noncontinuous soil (CK0), the abundance of *Actinobacteria* in the CK1 and T treatments significantly decreased (*P* < 0.05) by 39.56% and 35.31%, respectively, while the relative abundance of *Planctomycetes* significantly increased (*P* < 0.05) by 65.74% and 43.83%. The relative abundances of *Proteobacteria* and *Bacteroidetes* in the T treatment were significantly increased (*P* < 0.05) by 29.76% and 65.72% compared to those of CK1, respectively. However, lower relative abundances of *Acidobacteria*, *Gemmatimonadetes*, and *Planctomycetes* were observed in the T treatment, and they were equivalent to decreases of 38.13%, 35.50%, and 13.22%, respectively, compared to those observed with CK1 (Fig. 4G).

An average of 194,944 ± 25,340 fungal sequence reads per sample were obtained. The coverage data showed that 99.22% to 99.28% of the fungal taxa in the soil samples were detected (Fig. 4D). Decreases in the Chao1 and ACE indices were observed, indicating that the fungi changed from a peak richness state (CK1) to a low richness state (T) (Fig. 4E and F). At the phylum level, the fungal community was dominated by members of *Ascomycota* and *Zygomycota* (Fig. 4H). The most noticeable difference between continuous cropping soil (CK1, T) and noncontinuous soil (CK0) was the significant reduction (*P* < 0.05) in the relative abundances of *Glomeromycota* by 99.88% and 99.92%, respectively, and the significant increase (*P* < 0.05) in *Zygomycota* by 2.89 and 2.74 times, respectively. The abundances of *Ascomycota* and *Basidiomycota* in the T treatment differed significantly (*P* < 0.05) from those in CK1 and were equivalent to a 1.52-fold increase and a 92.53% decrease, respectively. Thus, the *Ascomycota* community was markedly promoted, whereas the *Basidiomycota* community was inhibited, in the presence of C3. At the genus level, the dominant fungi *Mortierella*, *Conocybe*, *Chaetomium*, and Fusarium showed a significant change (Fig. 4I). *Conocybe* was not found in the noncontinuous soil (CK0), but it was detected in the CK1 and T treatments, whereas the relative abundance of *Mortierella* increased significantly (*P* < 0.05) in the CK1 and T treatments. Compared with CK1, *Chaetomium* significantly increased (*P* < 0.05) by 6.49 times, whereas Fusarium significantly decreased by 84.61% in the T treatment.

In general, the bacterial and fungal OTUs in the continuous cropping soil (CK and T) were lower than those in the healthy soil (CK0) (Fig. S5). After the C3 treatment, the bacterial OTUs of continuous soil increased while the fungal OTUs decreased. The unique bacterial OTUs treated by T were 90.00% higher than those of the CK1. The shared OTUs of the CK1 and T fungi accounted for 68.47% and 78.94% of the respective OTUs, respectively, indicating that the fungal composition was different between the two treatments. After the C3 treatment, the characteristic OTUs decreased from 31.53% (CK1) to 21.06% (T).

### Actual effect of C3 on preventing and curing continuous cropping obstacles of melon.

**FIG 5:**
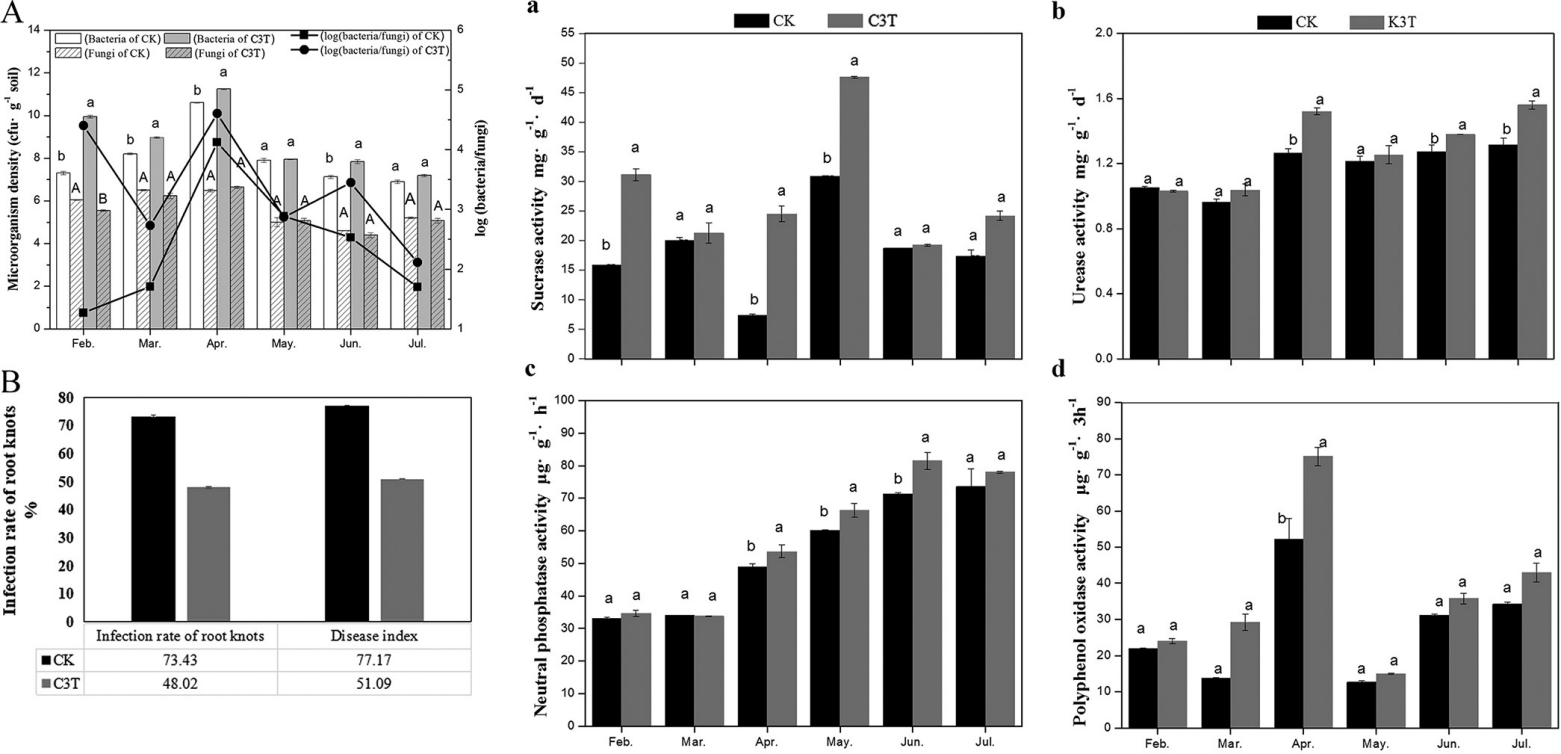
The effect of C3 on the soil microorganism densities, RKN infection rate, and activity of soil enzymes. (A) Microorganism density. (B) Infection rate of root knots. (a) Soil sucrase activity. (b) Soil urease activity. (c) Neutral phosphatase activity. (d) Soil polyphenol oxidase activity.

Bacterial and fungal microbial densities are shown in Fig. 5A. The levels of soil bacteria significantly increased (*P* < 0.05) during the whole growth period of melon after the inoculation of the C3 agent, except in May. The soil fungi density in the C3 treatment was lower than that in the CK treatment, although significant decreases were not observed, except in February. In the root zone soil of melon, the bacteria-to-fungi ratio was higher for the C3 treatment than for the CK treatment. Compared with CK, the number of root knots in the C3T treatment was reduced (Fig. S6), the RKN infection rate was significantly reduced (*P* < 0.05) by 25.41%, and the disease index was reduced to 51.09% (Fig. 5B).

The soil enzyme activity in the C3T treatment increased substantially compared with the CK treatment throughout the melon growing period (Fig. 5). The sucrose activity (Fig. 5a) of the C3-inoculated soil was significantly higher (*P* < 0.05) in February, April, and May, which were equivalent to increases of 96.11%, 234.15%, and 54.16% compared with that of the CK, respectively. Higher urease activity was observed in the C3T treatment group. The urease activities (Fig. 5b) in the C3T treatment were significantly increased (*P* < 0.05) by 20.06%, 8.58%, and 18.62% in April, June, and July compared to that of the CK, respectively. Neutral phosphatase activity (Fig. 5c) increased with melon growth, with the lowest and highest values appearing in February and June, respectively. After inoculation with C3, significant increases (*P* < 0.05) in the neutral phosphatase activity of 9.84%, 10.31%, and 14.12% were found in April, May, and June compared with that of the CK treatment, respectively. Increases in polyphenoloxidase (PPO) activity (Fig. 5d) were parabolic and reached a maximum in April, and the PPO activity in the C3T treatment was significantly higher (*P* < 0.05) than that of the CK treatment in April, resulting in a 43.77% increase.

Soil inoculation with the C3 agent significantly improved the fruit quality of continuously cropped melon (Table S6). Single fruit quality increased by 8.91%, although this increase was not significant (*P* > 0.05) compared that of the CK. The amounts of soluble sugar, soluble protein, and soluble solids were significantly increased (*P* < 0.05) by 28.79%, 48.09%, and 17.69%, respectively, compared with those of the CK. The fruit yield was significantly increased (*P* < 0.05) by 24.85% under the C3T treatment compared with that of the CK treatment.

## DISCUSSION

**FIG 6:**
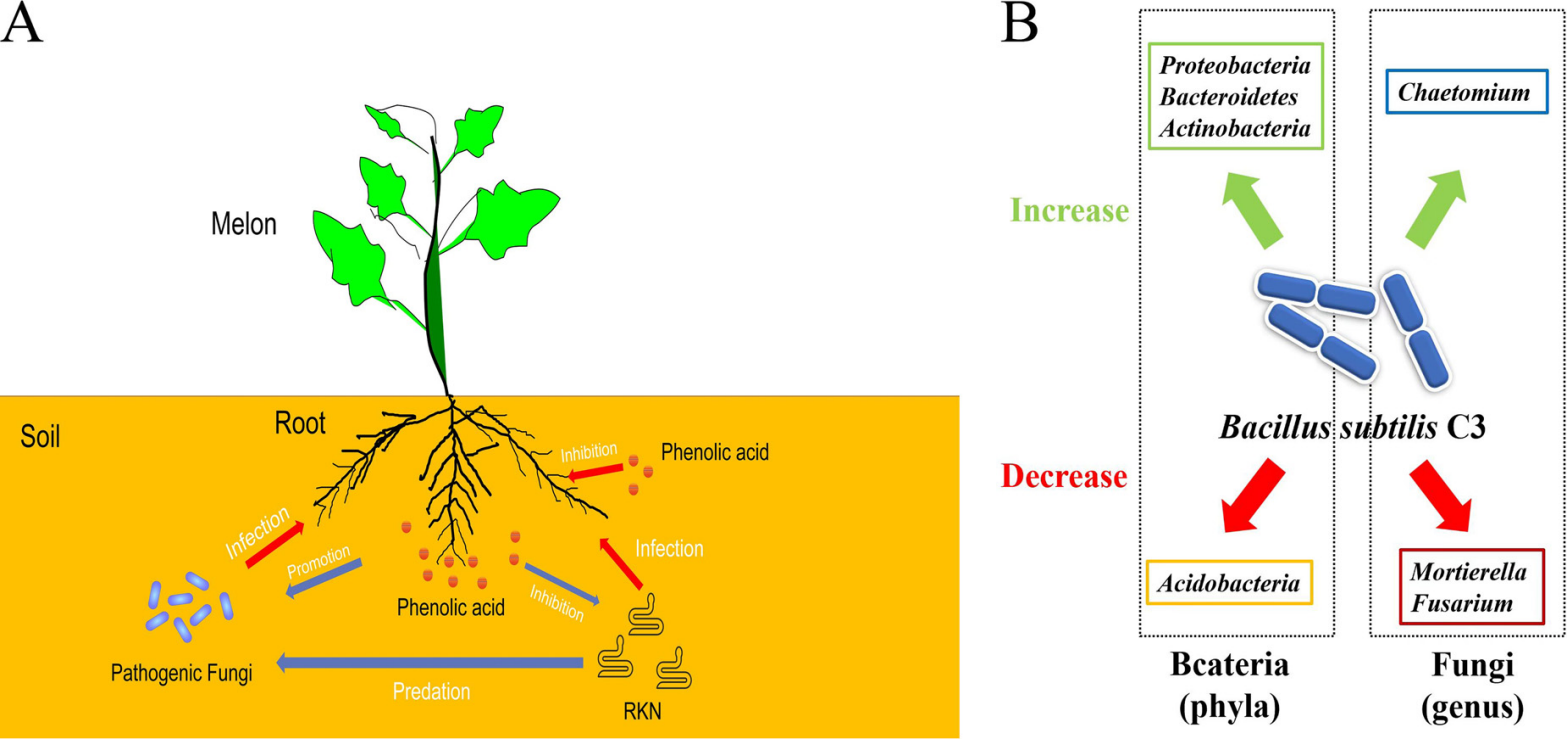
Model of occurrence of continuous cropping obstacles in the melon root system and the C3 prevention and control mechanism. (A) Model of occurrence of continuous cropping obstacles in the melon root system. (B) C3 prevention and control mechanism.

The occurrence of plant continuous cropping obstacles is mainly due to the accumulation of phenolic acids and increases in the number of pathogenic fungi and RKNs in the soil that are caused by the continuous planting of the same crops (26, 27). We have known that any two of these factors interact with each other, and our research continued on this basis and explored the relationships among phenolic acids, RKNs, and pathogenic fungi in continuous-cropping melon soil as well as their effects on melon growth after co-inoculation. We found that the pairwise inoculation of them had weaker inhibition on the growth of melon seedlings than did separate inoculation. This may be caused by the pairwise mutual restrictions. However, the combined inoculation of the three had the strongest inhibition, indicating that RNKs, HA, and Fusarium balance each other and coexist to inhibit the growth of melon. Therefore, we proposed for the first time that the vicious cycle (shown in Fig. 6A) in continuous cropping soil affects the growth of melon. CA, FA, and HA promoted the growth of Fusarium mycelium and indirectly increased the number of RKNs (RKNs preyed on Fusarium hyphae), and they could also directly inhibit the growth of RKNs and reduce their numbers.

Biological control technology security is important for promoting the sustainable and rapid development of agriculture. Bacillus subtilis is one of the most common biocontrol strains, has obvious effects on the prevention and control of various crop diseases and insect pests, and leads to the improvement of soil conditions and plant growth (28–31). In this study, Bacillus subtilis C3 was selected to prevent melon continuous cropping obstacles, and the results showed that C3 antagonized pathogens and RKNs as well as degraded self-toxic substances in the soil of continuously cropped melon. The vicious cycle of continuous cropping soil was weakened from three nodes. With an in-depth understanding of the degradation of phenolic acids by microorganisms, the use of microorganisms to decompose phenolic acids to eliminate continuous cropping obstacles is gradually receiving the attention of experts and scholars. When the phenolic acid in the soil reaches a certain concentration, bacteria with the ability to utilize phenolic acid were recruited to the rhizosphere soil. They consumed the mixture of phenolic acids as a carbon source, thereby reducing the growth-inhibitory effect of the phenolic acids on the plants (32). In this study, C3 degraded HA as a carbon source for its own growth. In the C3 and Fs3 coculture experiments, C3 inhibited the growth of mycelium by inhibiting spore germination, destroying the cell structure, and promoting mycelial cell apoptosis. Previous related studies have also achieved similar results. The treatment of Fusarium solani with Bacillus subtilis SPB1 lipopeptide lysed the mycelium and inhibited spore germination (33). Bacillus subtilis IB permeabilizes and disrupts Fusarium graminearum hyphae by producing fengycin (34). After inoculation with Bacillus subtilis C3, the number of root-knot nematodes in the soil was significantly reduced. It may be that the secondary metabolites of biocontrol bacteria restrict the growth of RKNs or that the addition of biocontrol bacteria increases the resistance ability of crops (35–37). Bacillus subtilis C3 antagonized pathogens and RKNs, decomposed self-toxic substances, and provided more favorable conditions for the soil environment of plant growth.

Phenols, such as benzoic acid and p-coumaric acid, may not directly poison plants, but they can indirectly affect their growth by changing the soil microbial community (27, 38, 39). RKNs are common in continuous crops, and they affect the activities of plant-pathogenic microorganisms and beneficial microorganisms in the rhizosphere by changing the quality and quantity of root exudates (40, 41). Therefore, improving the soil microbial community structure is another means by which to alleviate continuous cropping obstacles. In this study, C3 improved the composition and structure of the rhizosphere microbial community of continuously cropped melon soil. After the inoculation of C3 in the melon continuous cropping soil, the richness index of the bacterial OTUs increased while the richness index of the fungal OTUs decreased. This result is similar to those in previous studies, which show that bio-organic fertilizers change the structure of the soil microbial community (42–44). This may be due to the addition of C3, which affects the diversity of soil original microorganisms via competition or via its secondary metabolites. Thus, the addition of C3 strongly stimulated or inhibited specific groups of bacteria and fungi (shown in Fig. 6B). Many strains of *Actinobacteria* and *Proteobacteria* are common biocontrol strains (45), and their abundance in continuously cropped soil was significantly reduced compared with that observed in healthy soil; however, their abundance increased after applying C3 (46). This indicated that the addition of C3 promoted the growth of biocontrol strains. Studies have shown that the relative abundance of *Acidobacteria* has a negative correlation with the soil pH (47). The results of this study showed that in the continuous monoculture soil, C3 increased the soil pH by decomposing the phenolic acid and then decreased the relative abundance of *Acidobacteria*, which is consistent with the above results. For the fungal community, the long-term planting of melon changed the soil environment, and then the fungi of the genus *Conocybe* appeared. *Mortierella* is a kind of pathogenic fungus that can destroy plant health (48, 49), and its abundance in the melon continuous cropping soil increased significantly but decreased after the addition of the C3 strain, indicating that the addition of C3 can limit the growth of this fungus. Interestingly, under the T treatment, the relative abundance of Fusarium significantly decreased by 84.61%, whereas that of *Chaetomium* significantly increased by approximately 6.49-fold. *Chaetomium* spp. are widely distributed in natural soil and have the potential ability to suppress many plant pathogens and root nematodes (50). In contrast, Fusarium is a notorious pathogenic plant fungus that has a wide variety of hosts and infection strategies (51). Accordingly, C3 acts as a biocontrol agent and as a soil guard to maintain a healthy soil microbial structure by promoting the growth of beneficial bacteria and inhibiting the propagation of pathogenic fungi (Fig. 6B).

Reports have shown that plant growth promoting bacteria improve soil enzyme activity (52–54), such as sucrose, urease, neutral phosphatase, and PPO, among others. The results of this study also showed that the C3 treatment significantly increased the urease, neutral phosphatase, and PPO activity in the continuously cropped melon soil, which thereby improved the soil nutrient content. At the same time, the addition of C3 reduced the incidence of root-knot nematodes in continuously cropped melons and improved the yield and quality of the melons. These results show that the inoculation of C3 can effectively alleviate continuous cropping obstacles.

### Conclusions.

Phenolic acid, Fusarium, and RKNs all inhibited the growth of melon, and there was an interaction between them. The melon pathogen Fusarium solani (Fs3) has been used as a food to cultivate RKNs in large quantities. Phenolic acid promoted the growth of Fs3 hyphae and had various degrees of toxicity to the RKNs. The checks and balances among the three formed a vicious cycle that simulated the continuous cropping soil environment and led to the greatest inhibiting effect on the growth of melon. Bacillus subtilis C3 weakened the vicious cycle by inhibiting the growth of pathogenic Fusarium, decomposing phenolic acids, and reducing the number of nematodes. Simultaneously, C3 promoted the reproduction of beneficial microorganisms and inhibited the growth of pathogenic fungi, thereby improving the structure of the soil microbial community. The actual effect of C3 in alleviating the obstacles of continuously cropped melon was remarkable.

## MATERIALS AND METHODS

### Bacterial strains and RKNs.

Bacillus subtilis C3 (GenBank accession number KY983582) was isolated, identified, and stored in Cuiling Cao’s laboratory, and previous studies found that this strain could control the root rot of kiwifruit (25) and bulb rot disease in Fritillaria taipaiensis P.Y.Li. (55). The effective viable RKNs were taken from the roots of melons that were infected with RKNs in the greenhouse of the Yangling Zhonglai Agricultural Plantation Cooperative in Shaanxi, China.

### Isolation, identification, and reinoculation experiment with pathogenic fungi in the rhizosphere soil of continuously cropped melon.

In April of 2019, 10 g of soil were taken from the rhizosphere of melon after continuous cropping for 3 years in the greenhouse of the Yangling Zhonglai Agricultural Plantation Cooperative in Shaanxi, China (the initial soil conditions are provided in Table S1), and 90 mL of sterile water were added and shaken at 180 rpm for 30 min to prepare a soil suspension. The above suspension was coated on Rose Bengal Chloramphenicol Agar plates via the dilution plate coating method, and after culturing at 30°C for 2 to 3 d, single colonies were picked and purified twice on PDA solid medium. After the purified fungus was cultured on PDA solid medium for 10 days, the morphology of the strain was observed. The hyphae were picked up on a glass slide, stained with lacto phenol cotton blue solution for 5 min, and placed on a microscope (Olympus BX53) to observe the hyphal and spore morphology. The genomic DNA of the isolated fungi was extracted using a fungal DNA extraction kit, and the universal primers ITS1 and ITS4 (ITS1 is 5′-TCCGTAGGTGAACCTGCGG-3′, and ITS4 is 5′-TCCTCCGCTTATTGATATGC-3′) were used to amplify the ITS sequence. Agarose gel (1%) electrophoresis was used to detect the results of the polymerase chain reaction (PCR) amplification and recover the PCR product before it was sent for sequencing. The resulting sequence was uploaded to the National Center for Biotechnology Information (NCBI) GenBank and used for homology alignment. The obtained data were analyzed by the neighbor-joining method using MEGA7, and a phylogenetic tree was constructed via a bootstrap analysis with 1,000 repetitions (55).

Melon seeds (Zaomi No.1) were sterilized with 1% NaClO for 5 min and 75% ethanol for 5 min and then rinsed 3 times with sterile water. The seeds were placed evenly in a petri dish, covered with moistened gauze, and germinated at 30°C. After the seeds showed their white buds, they were then sown in substrate soil (Danish Pinstop) to raise seedlings. Many studies have found that the main pathogenic fungus of melon wilt and root rot is Fusarium (56–58). Therefore, our next research content mainly focuses on Fusarium. 4 strains of Fusarium were isolated here and individually cultured on PDA solid plates for 7 days. The spores were rinsed and collected with 10 mL of sterile water. Then, they were diluted to 10^6^ spores/mL for further use. Pot experiments were carried out in a greenhouse in the North Campus of Northwest A&F University (25°C, 16/8 h day/night cycle) on May 23, 2019, and the culture conditions of all of the pot experiments in this study were consistent. Blank soil (Table S1) without melon planting (the previous crops were wheat) was collected and put into pots with a weight of 1 kg/pot (the water content is about 20%, the same below). When the melon seedlings were grown to the one-and-a-half-leaf stage, seedlings with uniform growth were chosen and transplanted into the pots for the pot test. Every treatment had 10 pots. Three days after transplanting, the roots of the melon seedlings were inoculated with 10 mL of diluted Fusarium spores per each plant, and the control (CK) was inoculated with an equal amount of sterile water. After 30 d, the disease and growth status of the seedlings was observed.

### Interaction of Fusarium, phenolic acids, and RKNs.

**(i) RKNs cultured in vitro with Fusarium.** Roots infected with RKNs (the roots of melons with cucurbit-shaped and light-yellow root knots) were collected from the greenhouse of the Yangling Zhonglai Agricultural Plantation Cooperative. The roots were first soaked in 75% alcohol for 5 min, and then they were sterilized with 1% NaClO for 3 min, rinsed 3 times with sterile water, and cut into 0.5 cm long pieces. Then, they were incubated with a modified Baermann funnel method for 24 h to obtain an aqueous solution that contained the second instar larvae. The above solution was centrifuged at 2,000 rpm for 5 min to collect RKNs, and then a suspension of approximately 50 RKNs/100 μL was prepared. Then, 4 strains of Fusarium were inoculated onto PDA media. Each strain was inoculated onto 6 plates and was cultured at 28°C for 7 d. The thickness of the medium was approximately 5 to 10 mm to extend the culture time of the RKNs. The prepared RKN suspension was inoculated into the center of the double medium (PDA solid medium with Fusarium cultured for 7 d), and each plate was inoculated with 200 μL (approximately 100 RKNs) of suspension. The lid was closed, and the plates were allowed to stand for 2 h to allow the droplets to sink before further culture at 28°C for 21 d (16). The plates with the double medium were slightly tilted to the side, and 1 mL of sterile water was added at the tallest point. The plates were allowed to stand for 5 min to allow the sterile water to slowly flow down to collect the RKNs. Then, the suspension was centrifuged at 2,000 rpm for 5 min, and the Fusarium spores in the supernatant were removed to obtain an RKN suspension.

**(ii) Effect of phenolic acids on the mycelial growth of Fusarium.** Coumaric acid (CA), ferulic acid (FA), *p*-hydroxybenzoic acid (HA) with a self-toxic effect on melon, and the highly pathogenic Fusarium Fs3 were chosen for use in follow-up studies (59, 60). CA, FA, and HA were dissolved in a small amount of ethanol to prepare the required concentrations with sterile water. After passing through a 0.22 μm filter membrane, solutions were added to potato dextrose broth (PDB) medium at final concentrations of 5, 10, and 20 mg/L with an equal amount of sterile water added to the control. 5 plates per treatment were set up as replicates. Plugs of 6 mm in diameter with uniform growth at the edge of 7-day-old Fusarium colonies were inoculated onto PDB medium that had been treated with phenolic acids. One plug was used per plate, and the plates were incubated at 28°C for 7 d. After filtering the mycelium with filter paper, it was dried and weighed. The promotion rate was calculated according to the formula below.

$$\text{Promotion rate }\left(\%\right)=\frac{\mathrm{Treatment dry weight of mycelium - Control dry weight of the mycelium}}{\mathrm{Dry weight of the mycelium of the control group}}\times 100$$

**(iii) Effect of phenolic acids on RKNs.** After being dissolved in a small amount of ethanol, CA, FA, and HA were formulated into the required concentrations with sterile water (25, 50, 100, 200, and 400 μg/L; 25, 50, 100, 200, and 400 mg/L). Then, the phenolic acids were added to 96-well plates with 200 μL per well, using distilled water as a control. To each well, 20 μL of a nematode suspension (approximately 100 RKNs) was added. The culture plate was incubated at 28°C, and the RKN mortality and adjusted RKN mortality were calculated after 48 h using Formulas (1) and (2), which are given below.

(1) $$\mathrm{Mortality rate}\left(\%\right)=\frac{\mathrm{Number of dead nematodes}}{\mathrm{Number of tested nematodes}}\times 100$$

(2) $$\mathrm{Adjusted mortality rate}\left(\%\right)=\frac{\mathrm{Treatment nematode mortality - Control nematode mortality}}{\mathrm{1 - Control nematode mortality}}\times 100$$

**(iv) Effects of HA, Fusarium, and RKNs on the growth of melon seedlings.** HA was selected for its best growth promoting effect on Fs3 mycelium and its most toxic effects on melon RKNs. The RKNs were obtained by artificial cultivation on Fs3 double media. The soil, germination and transplanting methods were the same as those used in the aforementioned reinoculation test of the pathogenic fungi. The inoculum volume of Fs3 was 10 mL/plant (10^6^ spores/mL), and the inoculum volumes of the HA and RKNs were 400 μg/kg soil and approximately 2,000 RKNs/plant, respectively. The treatments of the experiment included: none of the RKNs, HA, and Fs3 were inoculated so as to have a control (CK); RKNs, HA, and Fs3 were treated separately (RKN, HA, and Fs3); and mixtures of RKN, HA, and Fs3 treatments (Fs3 + HA, RKN + Fs3, RKN + HA, and RKN + Fs3 + HA). There were 8 treatments in total, and each treatment was repeated 10 times. RKN, HA, and Fs3 spore suspensions were applied while the seedlings were being transplanted. After transplanting, the seedlings were grown for 25 d, and various morphological indices were measured.

### The mechanism by which C3 prevents continuous cropping obstacles in melon.

**(i) Decomposition and utilization of phenolic acids by C3.** CA, FA, and HA, at concentrations of 100 μg/mL, were separately added to MB solid medium (NH_4_NO_3_, 1.0 g; MgSO_4_·7H_2_O, 0.5 g; (NH_4_)_2_SO_4_, 0.5 g; KH_2_PO_4_, 0.5 g; NaCl, 0.5 g; K_2_HPO_4_, 1.5 g; agar, 18 g; distilled water, 1,000 mL) before solidification, and the medium was poured into petri dishes (60 mm diameter). A control with an equal amount of sterile water was established. C3 activated on Luria-Bertani (LB) medium was placed at the center of the MB medium with an inoculation needle and cultured at 30°C for 7 d. Each treatment was repeated 5 times.

**(ii) Inhibitory effect of C3 on the growth of Fusarium hyphae.** The prepared modified PDB medium (potato, 200.0 g; dextrose. 20.0 g; beef extract, 5.0 g; (NH_4_)_2_SO_4_, 1.0 g; MgSO_4_, 1.0 g; KH_2_PO_4_, 0.6 g; distilled water, 1,000 mL) was separated into 50 mL conical flasks with 20 mL per flask. C3 was cultured in LB liquid medium at 180 rpm at 30°C for 24 h to obtain its fermentation broth (the effective number of viable bacteria was about 3.01 × 10^10^ CFU/mL). C3 fermentation broth was inoculated into the flasks at 10% of the inoculum, and Fusarium hyphae were also picked and inoculated into the PDB medium. The control was not inoculated with C3 fermentation broth. Each treatment was repeated 3 times. Flasks were incubated at 28°C with shaking at 180 rpm for 24 h and left to stand at 28°C for 5 d. The mycelium was filtered, dried at 50°C, and weighed.

The hyphae of Fs3 were picked from liquid cocultivation after 8 and 24 h, and the morphology of the mycelia was observed via lacto phenol cotton blue staining. For the fluorescent staining, mycelium was placed in sterile 1.5 mL centrifuge tubes with 20 μL of 50 μg/mL propidium iodide (PI) and was incubated at 37°C in the dark for 15 min. A fluorescence microscope (Olymous BX53) was used to observe the staining of the mycelial cells. The Hoechst 33258 staining method employed was the same as described above. The concentration used was 10 μg/mL (55).

**(iii) Effect of C3 on RKNs.** The soil, germination, and transplanting methods were the same as the aforementioned reinoculation test of pathogenic fungi. There were three treatments here, including a control group (CK, 10 mL of sterilized LB liquid medium), an inoculation with RKNs (RKN, 10 mL of sterilized LB liquid medium + RKNs), and a combined inoculation of 10 mL C3 fermentation broth and RKNs (C3 + RKN). 10 pots were set up for each treatment as replicates. The C3 agent was mixed into the soil before the melon seedlings were transplanted. RKNs were obtained via the artificial cultivation of Fs3 and used to inoculate the melon roots (approximately 150 RKNs/plant) with a syringe 2 d after transplanting (on November 5, 2019). After 40 d, the biomass and agronomic traits were determined. 50 grams of fresh soil were weighed, and 60 mL of sterile water were added to it. After shaking and standing for 3 to 5 min, the supernatant was passed through 0.096 and 0.054 mm sieves. The filter residue on the 0.054 mm sieve was collected, and the volume was adjusted to 3 mL. After shaking, 50 μL of the above suspension was drawn and counted under a microscope. Fresh soil samples were taken to determine the water content. The number of RKNs was the number of nematodes per 100 g of dry soil.

**(iv) Effect of C3 on the microbial community in the continuously cropped soil of melon.** 2 soils were used in this pot experiment. The first soil was the aforementioned blank soil. The second soil was the continuous cropping potting soil collected from the root area of melons planted for the third time (10 to 20 cm from the taproot). Its characterization is shown in Table S1. 3 treatments were included: CK0, 0.5 kg of blank soil; CK1, 0.5 kg of continuously cropped potting soil; and T, 0.5 kg of continuously cropped potting soil and 5 mL of C3 fermentation broth added to each pot with sufficient mixing. Every treatment had 10 replicates in greenhouse in the North Campus of Northwest A&F University (25°C, in a 16/8 h day/night cycle) on November 5, 2019. After transplanting, the seedlings were grown for 30 d, and the rhizosphere soil of the potted seedlings was collected.

The soil samples were mixed thoroughly, transported in polyethylene tubes, and immediately placed in a −80°C refrigerator. The total genomic DNA of the soil was extracted from the soil using a PowerSoil DNA Isolation Kit (MoBio Laboratories Inc., Carlsbad, CA, USA). The gene-specific primers 515F and 806R were used to amplify the V4 regions of bacterial 16S rRNA genes (61), and the primers ITS1F and ITS2 targeted the ITS1 region of the fungal internal transcribed spacer (ITS). The purified amplicons were pooled in equimolar concentrations and employed for library construction. The final quality and concentrations of the libraries were checked using Agilent 2100 Bioanalyzer Instruments (Agilent Technologies Inc., Santa Clara, CA, USA). All library preparation was performed using an Illumina MiSeq Benchtop Sequencer (Illumina, San Diego, CA, USA) platform at Genesky Biotechnologies Inc. (Shanghai, China). The high-quality sequences were analyzed using QIIME standard operating procedures, and any reads that were 50 bp in length or contain unresolved nucleotides were removed (62). Operational taxonomic units (OTUs) at 97% similarity were identified using a UPARSE v7.0.1090 (63). The taxonomic identification of bacteria and fungi was performed against the Silva (64) and UNITE reference databases (65), respectively, based on a naive Bayesian classifier. Alpha diversity was calculated using the observed OTUs, The Chao1 and ACE indices were used to compare the bacterial and fungal diversity across different compartments and treatments.

### Greenhouse experiment.

A plastic greenhouse situated in Lingwan Village in Yangling (34°16′N, 108°0′E) was used for these experiments from January 18, 2019, to July 2, 2019. The experimental area included an area with severe RKN disease. The experiment consisted of two treatments. CK was a control treatment without C3 inoculation, and C3T was a treatment with the inoculation of C3 fermented broth into the rhizosphere of melon. Fermented C3 was inoculated three times, respectively, at the early flowering stage of male flowers, late flowering stage of male flowers, and early stage of fruiting with 20 mL/plant, 40 mL/plant, and 40 mL/plant, respectively. To detect the microbial (bacteria and fungi) abundance in the rhizosphere soil, 2 g of the rhizosphere soil were taken from each treatment every month during the growth cycle. LB agar and Rose Bengal agar medium (66) were used to evaluate the populations of bacteria and fungi, respectively, via the dilution coating method. The soil was collected when the melon was harvested to determine the following soil enzyme activities. The sucrase activity was determined via 3,5-dinitrosalicylic acid colorimetry. The urease activity was determined by sodium phenolate colorimetry. The polyphenol oxidase activity was determined via pyrogallic acid colorimetry. The phosphatase activity was determined via the phenyl disodium phosphate method.

After the melons had been harvested, the heavyweight output of a single fruit was counted. 30 fruits were randomly collected to determine their quality. The Coomassie brilliant blue-G250 method was used for the determination of soluble protein. Molybdenum blue colorimetry was used for the determination of vitamin C contents. Anthrone colorimetry was used for the determination of soluble sugar contents. A handheld Abbe refractometer method was used for soluble solids (Shanghai Optical Instrument Co., Shanghai, China). The fruit nitrate nitrogen was measured via spectrophotometry (67).

Five complete roots were selected from each treatment to determine the total number of lateral roots and the number of infected lateral roots with root nodules. The infection rate (I, infection) was calculated according to Formula (1), which reflects the severity of the root damage of each plant and was divided into four levels (0, I = 0%; 1, I = 1% to 25%; 2, I = 26 to 50%; 3, I = 51 to 75%; 4, I = 76 to 100%). The disease index (22) and control effect were calculated according to Formula (2).

Infection rate (%) =  number of lateral roots infected by root knot nematodes/total number of lateral roots×100.

Root knot index (%) = Σ(disease grade× number of plants in corresponding grade)/(4×total number of surveyed plants)×100.

Control effect (%) = (control root knot index − treatment group root knot index)/control root knot index×100.

### Statistical analysis.

Microsoft Excel 2007 (Microsoft Corporation, USA) was employed to process the data. Statistical analysis was performed using SPSS 23.0 (SPSS, Inc., Chicago, USA). Differences were determined by a one-way analysis of variance (ANOVA) followed by Fisher’s least significant difference (LSD) test. Statistical significance was determined at *P* < 0.05.
