# Supplementary material for: Biological Control of Melon Continuous Cropping Obstacles: Weakening the Negative Effects of the Vicious Cycle in Continuous Cropping Soil
Source: Microbiol Spectr. 2022 Oct 27;10(6):e01776-22. doi: 10.1128/spectrum.01776-22 (PMC9769590; doi:10.1128/spectrum.01776-22)
Supplement: Supplemental file 1 — Tables S1 to S6 and Fig. S1 to S6. Download spectrum.01776-22-s0001.pdf, PDF file, 0.5 MB [file spectrum.01776-22-s0001.pdf]

Biological control of melon continuous cropping obstacles: weakening the negative effects of the vicious circle in continuous cropping soil

Yongli Ku[1], Wenqiang Li[1], Xueli Mei[1], Xiangna Yang[1], Cuiling Cao[1#], Huimei Zhang[2], Le Cao[3], Minglei Li[4]

[1]*College of Life Sciences, Northwest A&F University, Yangling, Shaanxi Province, China, 712100*

[2]*College of Horticulture, Northwest A&F University, Yangling, Shaanxi Province, China, 712100*

[3]*College of Environment and Life Sciences, Weinan Normal University, Weinan, Shaanxi Province, China, 714000*

[4]*Institute of Soil and Water Conservation, Northwest A&F University, Yangling, Shaanxi Province, China, 712100.*

Running Head: Bio-control of melon continuous cropping obstacles

[#]Address correspondence to Cuiling Cao, caocuiling@nwsuaf.edu.cn.

Yongli Ku and Wenqiang Li contributed equally to this work. Author order was determined on contributions of later submitted manuscripts.

Table S1 Soil physical and chemical properties in the text.

Treatment	Nitrate Nitrogen mg/kg	Ammonium Nitrogen mg/kg	Available Phosphorus mg/kg	Available Potassium g/kg	Organic Matter %	pH
Continuously cropped melon rhizosphere soil	24.25±5.63	19.41±2.33	33.2±0.6	176.7±8.9	0.88%	8.3±0.5
Blank soil (the previous crop was wheat)	30.43±1.73	36.20±4.18	17.4±1.5	111.2±11.3	1.5%	7.0±0.2
The continuous monocropping potting soil	21.34±2.09	18.81±2.52	31.1±0.5	189.7±10	0.77%	8.6±0.6

Table S2 Plant biomass and agronomic traits of melon seedlings inoculated with four strains of *Fusarium* inoculated after 30 d in the pot experiment

Treatments	Plant height (cm/plant)	Root length (cm/plant)	Leaves number (/plant)	Largest leaf area (cm^2)	Fresh weight (g/plant)	Dry weight (g/plant)
CK	17.78±1.12a	10.82±1.15a	5.8±0.4a	23.36±2.03a	2.769±0.316a	0.156±0.012a
Fs1	16.29±1.71a	8.82±0.83b	5.4±0.5a	22.39±2.51a	2.497±0.283b	0.155±0.018a
Fs2	16.63±1.19a	11.26±0.59a	5.7±0.5a	23.01±1.35a	2.576±0.133ab	0.155±0.014a
Fs3	10.74±1.05b	8.13±0.53b	4.6±0.6b	14.33±1.46b	1.185±0.116c	0.090±0.009b
Fo1	13.54±1.44c	10.18±0.82a	4.8±0.4b	15.98±1.37b	1.455±0.028c	0.109±0.010b

Mean data with different lowercase letters indicate a significant difference ($P < 0.05$). The same below.

Table S3 Calculation of the density of RKN artificially cultured in double medium of *Fusarium*

Strains	Density of RKN (Number/cm^3)
Fs1	4352±380b
Fs2	10757±301a
Fs3	9030±290a
Fo1	12863±826a

Table S4 Inhibitory effect of C3 on the growth of Fs3 after 5 d of co-cultivation

Treatments		Dry mycelial weight (mg)	Inhibition rate (%)
Fs1	Fs1	155.500±0.707a	63.754
	C3+ Fs1	56.000±1.414b	
Fs2	Fs2	118.667±2.517a	33.848
	C3+ Fs2	78.500±7.778b	
Fs3	Fs3	157.000±4.243a	37.580
	C3+ Fs3	98.000±9.899b	
Fo1	Fo1	82.500±6.364a	26.667
	C3+ Fo1	60.0500±9.192b	

Table S5 Effect of C3 on biomass and horticultural traits of melon seedlings after inoculation with RKN cultured by Fs3

Treatments	Plant height (cm/plant)	Root length (cm/plant)	Largest leaf area (cm^2)	Leaves number (/plant)	Shoot fresh weight (g/plant)	Root fresh weight (g/plant)
CK	12.7±0.7b	15.7±0.1b	60.82±7.40b	9.3±0.6b	12.146±1.410b	1.145±0.156a
RKN	12.9±1.1b	13.7±1.7b	57.11±4.00b	8.0±1.0b	9.738±0.232b	0.803±0.136a
C3+RKN	15.3±0.05a	25.1±5.9a	102.61±4.15a	12.4±0.6a	22.520±5.532a	1.411±0.585a

TableS6 Fruit quality and yield of infected melon in response to C3 infection

Treatments	Single fruit quality g	Soluble sugar mg/g	Soluble protein mg/g	Soluble solids %	Vitamin C mg	Yield kg/10 plants
CK	247.00±21.69a	120.89±8.11b	4.18±0.01b	13.17±0.29b	85.00±8.04a	7.296
C3T	269.00±19.86a	155.69±18.85a	6.91±0.52a	15.50±0.00a	96.29±6.90a	9.109
Increase rate()%	8.91	28.79	48.09	17.69	13.28	24.85

Fig. S1 Morphology of colonies, hyphae and spores of 8 fungi isolated from the soil of continuous

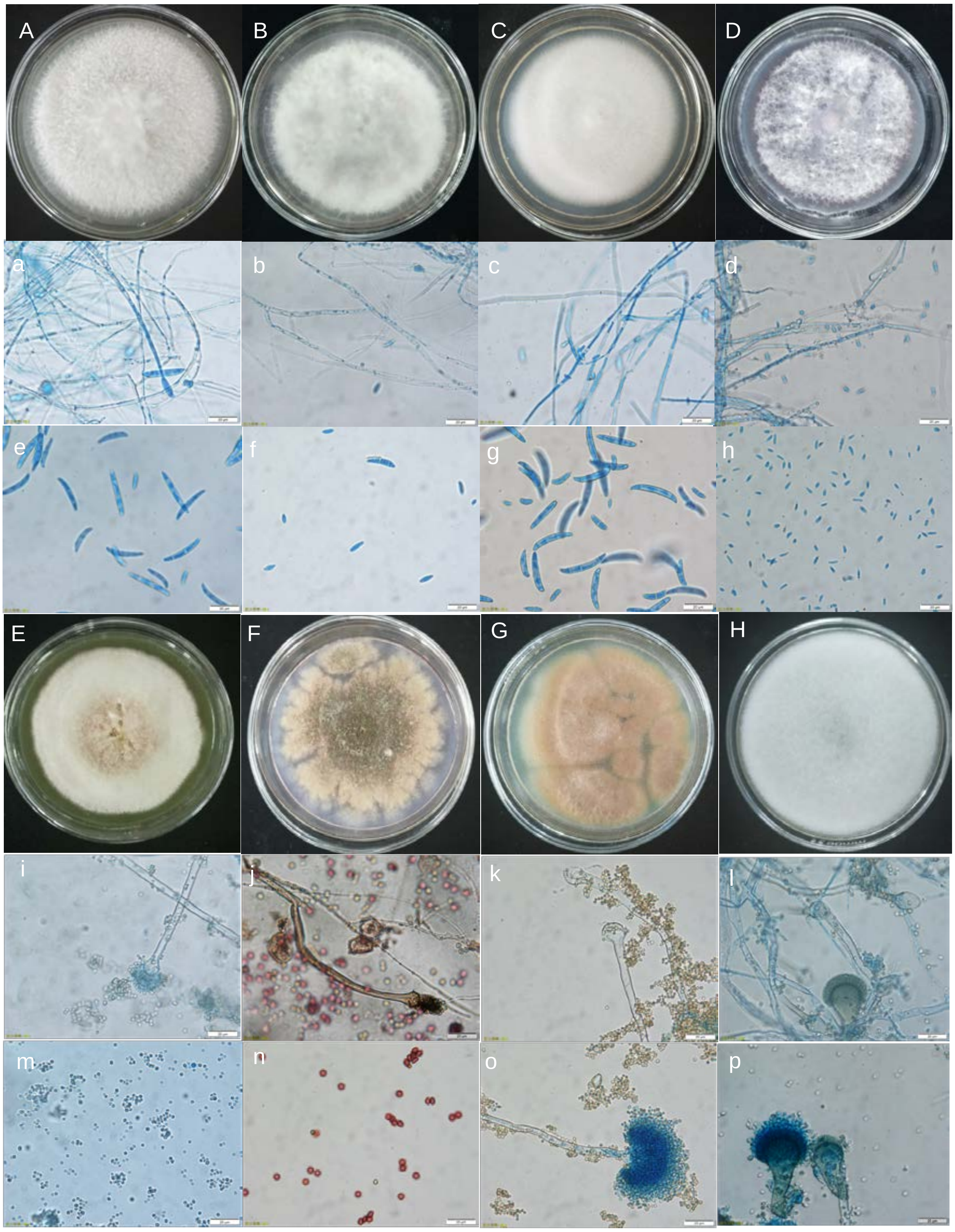

cropping melon. The morphological observation of hyphae and spores was stained with lacto phenol cotton blue (Bars =20 μm).

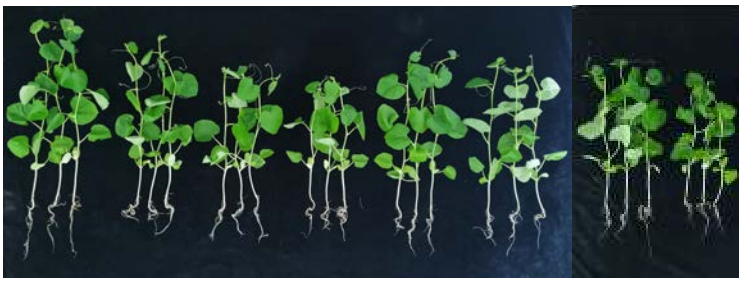

CK HA Fs3 RNK Fs3+HA RNK+Fs3 RNK+HA RNK+Fs3+HA

Fig. S2 Effect of K3 on the growth of melon inoculated with HA, Fs3, and RKN

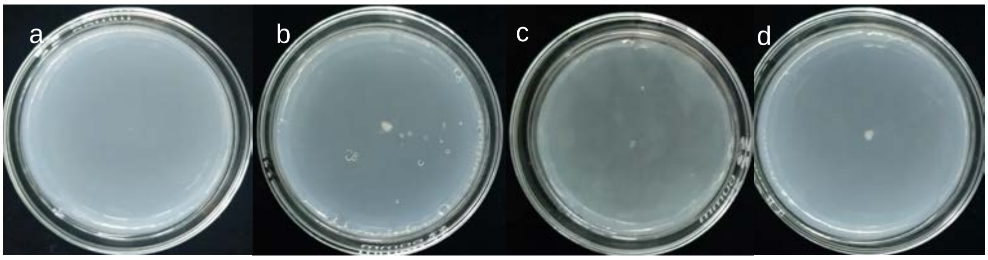

Fig. S3 Decomposition and utilization of phenolic acids with K3. a: Control; b, c, d: MB medium with CA, FA, and HA respectively as a carbon source inoculated with K3 after 7 d.

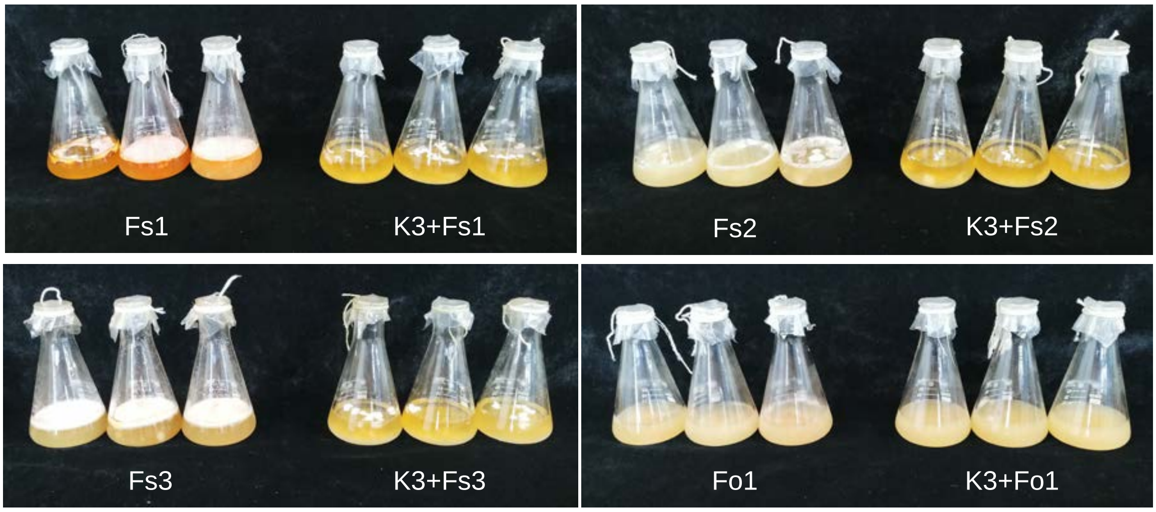

Fig. S4 Inhibitory effect of K3 on the growth of Fs1, Fs2, Fs3, and Fo1 hyphae after 5 d of co-cultivation

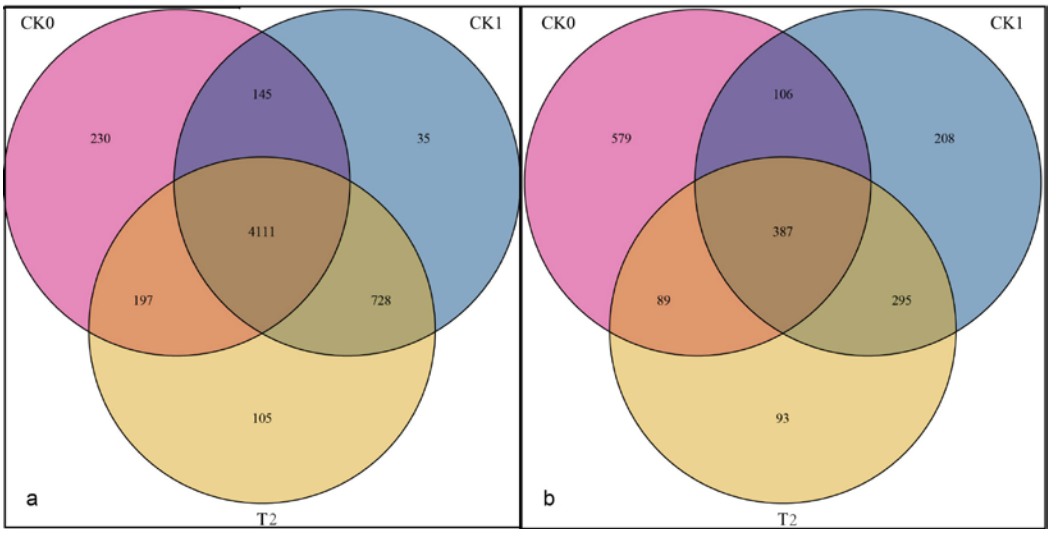

Fig. S5 OTU distribution Venn diagram. **A**, Bacteria. **B**, Fungi.

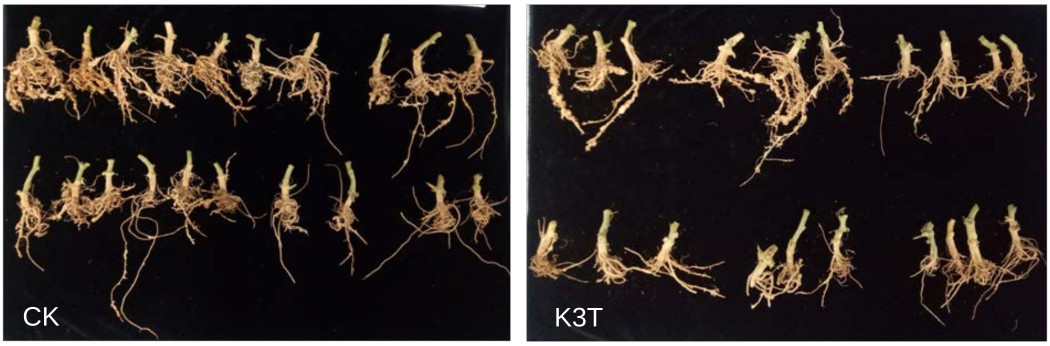

Fig. S6 Melon roots infested by RKN
